# Views about HIV/STI and health promotion among gay and bisexual Chinese and South Asian men living in Auckland, New Zealand

**DOI:** 10.3402/qhw.v11.30764

**Published:** 2016-05-20

**Authors:** Stephen Neville, Jeffery Adams

**Affiliations:** 1 Department of Nursing, Auckland University of Technology, Auckland, New Zealand; 2 SHORE & Whariki Research Centre, Massey University, Auckland, New Zealand

**Keywords:** Men who have sex with men, HIV, migrant, ethnic minority, Chinese, South Asian, gay, condom use, health promotion

## Abstract

Ethnic minority gay, bisexual, and other men who have sex with men (MSM) are considered to have a high risk for HIV infection. The aim of this study was to identify some of the ways Chinese and South Asian MSM talk about and understand issues related to HIV/STI and health promotion, as well as highlighting some of this group's health promoting behaviours. A qualitative study using face-to-face interviews with 44 Chinese and South Asian MSM living in Auckland, New Zealand, was undertaken. Following data analysis, four major themes were identified: *the importance of condoms*, *condom use*, *HIV/STI practices*, *and HIV health promotion*. The results showed that the men interviewed had a good understanding of the benefits of using condoms for anal sex. They also reported strong recall of the local HIV health promotion campaigns which seek to influence men's behaviours through promotion of a single, unequivocal message to always use a condom for anal sex. The men however did not always report consistent condom use, and a range of reasons why this happened were identified. Among the men who discussed testing practices, regular testing was much more likely to have occurred in men who have lived in New Zealand for more than 5 years. These results suggest that future health promotion initiatives should be tailored to ensure the needs of Chinese and South Asian MSM are appropriately addressed when promoting condom use for anal sex.

The global epidemic of HIV among gay and bisexual men continues to expand (Beyrer et al., 2012), with one of the key drivers being the continued criminalization of homosexuality in a number of countries (Human Dignity Trust & Commonwealth Lawyers Association, 2015). Globally, HIV infection rates are higher among gay, bisexual, and other men who have sex with men (MSM) than other population groups (Altman et al., 2012). Within MSM populations, migrant and other ethnic minority gay men are often considered to be a group at particular risk of acquiring HIV. A developing body of literature points to a number of issues around HIV for these men.

Migrant and ethnic minority men appear at particular risk of HIV infection post-migration (Fakoya et al., 2015). This is often theorized as arising from a vulnerability experienced by these MSM as they move to places with unfamiliar environments and sexual cultures, and to which they come armed with the attitudes and views and condom use practices from their home communities (Kobrak, Ponce, & Zielony, 2015). In a qualitative study of migrants from Central and Eastern Europe living in London, the process of migration was found to influence the sexual behaviours of the men. It was identified the men extricated themselves from the constraints of their home societies and this greater freedom along with greater access to gay venues resulted in increased sexual activity. High-risk sexual behaviour for these men arose from their “sexual mixing, the use of commercial sex and perceptions of risk in the UK vis-á-vis Central and Eastern Europe …” (Mole, Parutis, Gerry, & Burns, 2013, p. 86).

The published data about HIV prevalence among migrant ethnic minority groups are mixed, and prevent generalized comments about prevalence. In particular, even within one country prevalence may vary between migrant groups. For example, in a study of more than 12,000 men in the United Kingdom self-reported HIV seropositivity was very low for men of South Asian, Chinese, and “other Asian” ethnicity and for men born in Central or Eastern Europe but was found to be elevated for men born in South or Central America (Elford et al., 2012). In Australia, higher rates of HIV prevalence were found among migrants from low and middle-income countries than high-income countries (McPherson, McMahon, Moreton, & Ward, 2011). Higher prevalence of HIV for MSM has also been found among internal migrants within China (Mao et al., 2014).

HIV testing and counselling is a critical component of any HIV prevention efforts. This is usually achieved by increasing the uptake of HIV testing and decreasing the number of undiagnosed people with HIV (Hoyos et al., 2013), with some studies investigating testing practices among migrant and ethnic minority men. For example, in the USA a study of Latino MSM found testing rates were lower among both recent and established migrants than among US-born Latino MSM (Oster et al., 2013). Limited access to healthcare has been shown to be a barrier to adequate levels of HIV testing amongst Latino migrant men (Martínez-Donate et al., 2015).

To promote our understanding of the vulnerabilities of migrant and ethnic minority men, this article reports on a study among Chinese and South Asian MSM living in Auckland, New Zealand, with the aim of identifying some of the ways these men talk about and understand issues related to HIV/STI and health promotion, as well as highlighting some of this group's health-promoting behaviours. Gaining such knowledge we argue is crucial so that public health and health promotion initiatives can be responsive to the HIV prevention needs of these populations. More culturally specific tailored HIV prevention interventions are needed to ensure meaningful engagement from at-risk population groups.

## Methods

### Design

A qualitative descriptive research design was used for this study. Qualitative description enables researchers to interpret and describe events in everyday terms and is suitable for providing straight-forward answers to research questions without the researcher being constrained by a particular philosophical or theoretical framework (Sandelowski, 2000). The use of qualitative description recognizes that participants are best placed to describe their views about a topic from the context of their own experiences (Winters & Neville, 2012). The contextual and personal information gained through the use of a qualitative descriptive approach enables the researcher to gain rich, in-depth information about the phenomena being studied.

### Recruitment

The research aimed to recruit two broad groups of men: those Chinese and South Asian MSM who moved to New Zealand within the last 5 years, and those Chinese and South Asian MSM who were either born in New Zealand or moved to New Zealand more than 5 years ago. Multiple methods were used to recruit the men. Advertising and promotion was undertaken in a variety of ways including posters and business cards distributed at gay venues and events (e.g., One Night in Mumbai party), postings on Facebook and other webpages (e.g., Love Your Condom Facebook page), advertisements on gay dating apps (e.g., Grindr), and publicity in gay media (e.g., gaynz.com). All the promotion and advertising was designed to direct men to the research study's website which contained information about the study and provided information about and a photograph of each of the four interviewers. The interviewers were comprised of the two researchers and two casual interviewers (one identified as Chinese and the other was of South Asian descent). Potential participants were directed to contact their preferred interviewer to arrange an interview. In addition to this advertising, several Asian men who had experience recruiting gay and bisexual men for another study were engaged to recruit potential participants for this study. Recruiters approached men within their personal, social, or other networks to introduce the study. Interested potential participants were asked to select their preferred interviewer and provide their contact details to the recruiter. This information was passed on to the research team and the selected interviewer contacted the potential participant to arrange an interview.

### Participants

Forty-four men took part in the research (Table I). Equal numbers of men who moved to New Zealand within the last 5 years (<5 years) and those who were either born in New Zealand or moved to New Zealand more than 5 years ago (>5 years) took part in the research. Demographic details were provided by most, but not all, participants.

**Table I:** Research participants.

Chinese living in NZ <5 years		Chinese living in NZ>5 years	
13 participants (10 demographic profiles completed)		14 participants (8 demographic profiles completed)	
•	Place of birth: China, Malaysia	•	Place of birth: China, Malaysia, New Zealand, Hong Kong
•	Age range: 18–25 years (mean=21.7 years)	•	Age range: 19–28 years (mean=23.4 years)
•	Work/education: Student (9), work (1)	•	Work/education: Student (3), work (5)
•	Relationship status: Single (6), Partner (4)	•	Relationship status: Single (6), Partner (2)

South Asian living in NZ < 5 years		South Asian living in NZ > 5 years	

9 participants (9 demographic profiles completed)		8 participants (8 demographic profiles completed)	
•	Place of birth: India, Pakistan, Myanmar	•	Place of birth: Fiji, Sri Lanka, India, Pakistan, Singapore, Indonesia
•	Age range: 19–29 years (mean = 24.5 years)	•	Age range: 18–29 years (mean = 23.6 years)
•	Work/education: Student (8), work (1)	•	Work/education: Student (4), work (4)
•	Relationship status: Single (7), Partner (2)	•	Relationship status: Single (8)

### Data collection and analysis

Data were collected using individual face-to-face interviews (one interview was by telephone). Individual interviews provide an excellent way of accessing individual's personal accounts and hearing detailed narratives about their lives (Green, 1999; Reinharz, 1992). The majority of the interviews were conducted in English; however, three were undertaken in Mandarin. All interviews were audio recorded with the participants’ permission. Interviews were then professionally transcribed.

A general inductive approach was utilized to analyse the raw data produced from the interviews (Thomas, 2006). This approach begins with the transcription of the digital recordings into written form and ends with the creation of a set of categories. Both the research aims and the raw data guide data analysis (Thomas, 2006). Following transcription care was taken to ensure the transcripts reliably reflected the content of the interviews. All transcripts were printed, read independently, and discussed by both researchers to ensure that the content of all transcripts was understood. The creation of categories, also called themes, involved the development of upper level (derived from the research aims) and lower level (derived from the raw data) categories (Thomas, 2006). The process of developing the lower level or more specific categories began with multiple, close readings of the raw data. After closely reading the raw data generated from the interviews and with the research aims in mind, sections of the text that were of interest were separately and independently highlighted by both the researchers. These sections of text were then summarized onto a data file. The summarized text sections from each interview were then reviewed and agreed on by both the researchers and placed into categories. The categories resulting from the interview data were cross-compared, and common categories formed for all transcripts. A closer investigation of the categories revealed similarities as well as differences among them. These were grouped together as upper level categories and were reported as themes.

### Ethics

This research was conducted under the guidelines of Massey University, and the approval from the Northern Human Ethics Committee for the research was obtained in January 2014. All participants were fully informed about the study through a participant information sheet and any questions participants had were answered before the consent form was signed. The voluntary nature of participation in the study and the no-direct approach from the researchers minimized the risk of people feeling like they had been coerced into taking part in the research. Phone numbers for free counselling and other support agencies were provided to all participants in case they became distressed after taking part in the research, or if they wished to discuss any issues that were raised as part of the research interview.

## Results

This article focuses on the themes evident across the men's talk. These themes are identified as follows: (1) the importance of condoms, (2) condom use, (3) HIV testing practices, and (4) HIV health promotion.

### The importance of condoms

Traditionally, HIV health promotion and prevention efforts in New Zealand and elsewhere have encouraged MSM to use condoms for anal sex (Neville, Adams, & Holdershaw, 2014; Saxton, Dickson, & Hughes, 2014) as they are effective in preventing HIV transmission in MSM who have anal sex (Shernoff, 2005; Sullivan et al., 2012). Among the participants there was a very high awareness about the importance of MSM using condoms for anal sex, and all participants from all groups identified that condoms are vital to prevent HIV infection and acquiring an STI.

Yeah every time I must use a condom. I know about AIDS HIV, so I just told myself if you hook up you must play safe and use a condom. (Chinese <5 yrs)

I think kissing and cuddling is ok and if you have oral sex, if someone sucks you it is ok, but if you suck other people use a condom and be sure if you have anal sex you use a condom. (Chinese <5 yrs)

A few men also identified using lubricant when having anal sex was necessary. This view reflects the clinical guidelines for correct use of condoms (Centers for Diesase Control, 2013).

… and obviously using condoms is not enough, always make sure you have got enough lubricant and what not. (Chinese >5 yrs)

In addition to the recommended strategy of using condoms, some men identified other approaches for managing risk in sexual activities. For example, one participant noted that he would try to assess whether the other man had a sexually transmitted disease (infection) before having anal sex. Practices such as this, although flawed, have been noted in other population groups in New Zealand (Adams & Neville, 2009).

Safe sex means we use protective things and first like if I hook up with someone I just try to make sure he is clean and free from all STDs by how he looks and all those things. I make sure he wears a condom [for anal sex]. (South Asian <5 yrs)

In a similar vein, one participant reiterated the importance of using condoms but identified an expectation that men should disclose if they have HIV to their potential sex partners, presumably as one way to identify who to have to sex with. He also carefully considered the potential for stigma in this type of action.

I just think protection is very essential and protecting yourself is very vital for any sexual activity that you take part in. I do also think it is important that someone discloses their HIV status but then I do see the negative in that and the stigma in that as well actually. But I just believe if you are going to be involved in these sorts of sexual acts [anal sex] it is important to get the word out that you need to be protected [use a condom]. (South Asian >5 yrs)

There was acknowledgment among men that having sex in a safe way was a reciprocal responsibility demonstrating an ethic of care for the well-being of both partners.

Well in my part I think it's fairly easy because I'll be upfront, I'll be like … you know … because I have to protect myself but also to protect the other party as well. I will be upfront about it, I will definitely tell them like hey if we're going to do this, have you got condoms. (Chinese >5 yrs)

Similarly, a few men expanded on the notion of safe sex (using condoms for anal sex) and framed it more holistically. In this extract, for instance, aspects of sex needing to be consensual and both partners being comfortable about it were raised.

Well obviously like the condom part, but also like, you know it is important to make sure it is consensual … where both parties are quite comfortable about having sex. (South Asian >5 yrs)

### Condom use

The men's interview data highlighted that having a theoretical understanding of condom use as a “desired” safe sex practice did not always translate into practice. Several men reported having anal sex without condoms. The men described several scenarios when condoms may not be used consistently. Taken together these scenarios demonstrate the complexity around decision-making in relation to condom use as identified in other local and international studies (Adams & Neville, 2009, 2012; Neville & Adams, 2009). The first of these scenarios related to what the participant described as making a poor decision.

I pretty much do it [not use condoms] with people I know and trust I guess they could have HIV and not know about it … Like I have not always used a condom … I don't know why, I just didn't. Just a momentary decision and I just didn't make a very good decision but I can't do anything about it now. (Chinese <5 yrs)

Another man, the only one who reported being HIV-positive, noted that he looked for opportunities to have bareback sex, that is anal sex without condoms with other HIV-positive men. However, he did use condoms when having sex with men who are HIV-negative.

I use condoms if I'm playing with other negative people, or if I don't know their HIV status. Hence the reason why the bareback website is a good way of identifying with other positive men which I don't use condoms with. (Chinese >5 yrs)

The actions described in the two above excerpts provide examples of a fundamental issue in health education where having knowledge does not necessarily lead to “rational” action and to the adoption of the recommended health-promoting behaviours (Airhihenbuwa & Obregon, 2000; Nutbeam, 2000). It may however recognize that the men have other priorities which do not support health-promoting behaviours but which they see as preferable.

Several men spoke about a contested area of using condoms for anal sex with a boyfriend or life partner. Some men discussed how they had agreed with their partner not to use condoms for anal sex.

So I guess safe sex is about reducing the risk of transmission [HIV and STI]. Because I'd never had sex before I met my boyfriend … we've both been tested [negative] so we're pretty happy with that, so we decided not to, yeah [use condoms]. (Chinese >5 yrs)

Yeah I used condoms with everyone until I met [name of person]. Like neither of us had had much penetrative sex and condom use wasn't much of an issue because I just didn't have that much anal sex. But yeah once we'd started seeing each other we decided we wouldn't use condoms because of both our sexual histories there was no risk. That [decision] went with a strong requirement to talk about who we were seeing and what we were doing and stuff…. (South Asian >5 yrs)

These excerpts are examples of the concept of “negotiated safety.” This concept requires partners in a regular relationship who have tested HIV-seronegative to agree to have unprotected anal intercourse only with each other, while also having a negotiated agreement about sex that might take place outside that relationship (Elford, Bolding, Maguire, & Sherr, 2001; Kippax et al., 1997). In New Zealand, this strategy is not supported by current HIV health promotion initiatives.

Although these kinds of explanations for not using condoms indicate that the men had some agency and were making decisions that suited their circumstances, there were a few reports of men being “compelled” to engage in unwanted sex.

He didn't want to use condoms and I'm just really lenient. I was like ‘sure why not’. I didn't enjoy the sex at all because deep down I'm really afraid but I'm just not strong enough to say no. So I can't really enjoy the sex 100%. (Chinese <5 yrs)

I like to be safe, but once I had sex with a guy whom I met on Grindr, he didn't want to use condoms. I was very afraid to do that, but I just accepted because I thought he was clean [meaning HIV negative] and neat and we just had sex without a condom. That's why I just went for an HIV test. (South Asian <5 yrs)

What is clear is that these men did not have the individual resources to draw on to say no to this unwanted sex; and such descriptions are congruent with other research that highlight the vulnerability of some MSM to sexual coercion (Braun, Schmidt, Gavey, & Fenaughty, 2009; Braun, Terry, Gavey, & Fenaughty, 2009).

### HIV/STI testing practices

Across the groups there was considerable variability in testing for HIV and/or STIs with some participants testing regularly and others not testing at all. Regular testing for HIV/STI was much more likely to be reported by men living in New Zealand for 5 years or more: South Asian (5 of 8 men interviewed) and Chinese (4 of 14). Men who moved to New Zealand in the last 5 years were much more likely to have been tested overseas—with the most recent test often in relation to immigration processes.

I go like every 3 months [to be tested] but sometimes I had a worry [didn't use a condom] I would go sooner … I rush to it. The most recent time was 2 weeks ago when I went to Body Positive [a place where HIV testing is available]. (South Asian <5 yrs)

Yes one time here and before I come here there was once because I had to do a medical check-up. (Chinese <5 yrs)

This variability in testing practices supports results from sexual behaviour surveys in New Zealand that have shown Asian (along with Pacific) MSM are less likely to have had an HIV/STI check than men of other ethnicities (Dickson, Ludlam, Saxton, & Hughes, 2015; Lachowsky et al., 2014).

A few men described how having an HIV/STI test was not relevant to them because of their sexual practices. One of these men reported he used condoms for anal sex but still decided to be tested; while the other noted that he did not see the need as he only has oral sex.

I just test for the sake of being tested. I just test to make sure I don't have any disease [HIV or STI] but only occasionally. I had one done in June but I knew I would be fine because I had safe sex … So far the people I have met, they all agree to use condoms. (Chinese >5 yrs)

I have learned … I know about HIV and STI but I haven't checked myself for HIV because I am a virgin and I haven't been having anal sex, just oral sex. (South Asian <5 yrs)

Several men identified where they had an HIV test. The most commonly reported places were the Burnett Clinic (operated by New Zealand AIDS Foundation [NZAF], a not-for-profit organization providing HIV prevention programmes, HIV testing, counselling, and support services) and Body Positive (peer support and advocacy group for people living with HIV). Other men reported being tested at Auckland Sexual Health Services (a service of the local district health board). Only one of the men identified getting tested by their general practitioner (family doctor).

I went to NZAF to get tested. I always do it every year to make sure and my partner does that as well. I never did it back home because I was scared if I go people would know and see me. So when I first came here I went to the Burnett Clinic. (South Asian >5 yrs)

Yeah, I've actually done it quite a few times over the years with Body Positive, because they have that fast testing and I've actually been to them for a regular check-up kind of thing. Even with my GP we do it annually so yeah I test and I don't feel ashamed of it. (Chinese >5 yrs)

Several men described the barriers to testing including not knowing how to access an HIV test and difficulties discussing sexual health issues with their general practitioner.

When I was in China I had a test every half year … Yeah I don't have HIV test here in Auckland because I don't know where to find where I can do that. (Chinese <5 yrs)

I haven't had any sexual health check-ups so far and like I would love to go to a different GP [general practitioner] if I have to but I am just not comfortable discussing my sexual health with my current GP. (South Asian <5 yrs)

The identification of barriers to testing is not surprising, as there is good evidence from surveys of New Zealand MSM that many do not disclose their sexuality and/or sexual practices to their general practitioner (Ludlam, Saxton, Dickson, & Hughes, 2015; Neville & Henrickson, 2006). Many others adopt very considered and cautious practices in the management and disclosure of their sexuality with doctors (Adams, McCreanor, & Braun, 2008) and also more broadly with others including family, friends, and work colleagues (Adams, Braun, & McCreanor, 2014). Additionally, many men do not make links between their sexuality and their health needs (Adams, Braun, & McCreanor, 2012; Adams, McCreanor, & Braun, 2013).

### HIV health promotion

Nearly all participants recalled seeing one or both of the HIV health promotion campaigns (Get it On! and Love Your Condom) run by NZAF; although some men did require prompting. These high levels of awareness are in line with other research which shows nearly all (97%) MSM in a gay-community survey recalled seeing messages about condom use for gay and bisexual men, and of these men, 93% had seen at least three different messages (Adams & Neville, 2013). Several noted how very prominent these campaigns were; many of the messages seen were in public spaces such as on billboards and at bus stops, and online including Facebook.

Love your Condom and Get it On! it is just all around town … and also on the internet. When you search gay stuff it will pop up like Get it On! Love your Condom, sometimes on Facebook … yeah it is a good message. (Chinese <5 yrs)

Oh I have seen posters, I have seen T shirts … I actually ‘liked’ the [Facebook] page … And one of the things I really liked about LYC campaign was that they had posters in areas like Otara which was I think was really, really nice initiative because these are some of the areas where we need a lot of information about safe sex. (South Asian <5 yrs)

The men also understood the HIV health promotion message was about safe sex—that is, the use of condoms for anal sex. This understanding also reflected other studies that have identified high levels of understanding that the purpose of the messages is to promote condom use (Ludlam, Saxton, Dickson, & Hughes, 2012). These messages were identified as being clear, strong, and relevant.

You mean the two campaigns? Yeah it is very relevant. I used to see a lot of these posters on bus stops and things like that … It's really relevant especially for people from the gay community. (Chinese >5 yrs)

There was a mixed view about the message content—some men noted they were effective without being explicit; whereas others described the campaigns as highly and overtly sexualized. One concern was the campaign may contribute to negative views linking being gay to sex.

The one thing I haven't liked about it is it makes other people, like straight people think gay people are really promiscuous. (Chinese <5 yrs)

Another view was that a sexualized focus in the campaigns did not sufficiently acknowledge the importance of relationships (presumably over casual sex), which he identified as a feature of Asian culture.

I think it is about the culture because Asians are more into relationships. I mean more loyal to each other, but white guys, while not being racist, tend to be more active and promiscuous in sexual activities. I think condom advertising campaigns should get across the importance of relationships. (Chinese <5 yrs)

In other respects, the current health promotion activities were described as engaging well enough with Asian MSM. Some participants felt greater diversity including Chinese and South Asian men and different body types would enhance engagement with the messages. This was seen as best integrated into current campaigns and undertaken in a natural way without highlighting Asian men specifically.

I don't especially feel marginalised by the sort of information being given out but I don't strongly identify … it's a problem that a lot of these images and I guess a lot of the information assumes sort of like white and upper middle class sort of gay men, which is problematic. (South Asian >5 yrs)

I think we need to have a little bit more, well a lot more actually of Asian representation in campaigns like just the face would be good … because it will resonate much more within the community, with the gay Asian group. (Chinese >5 yrs)

Two men mentioned it would be useful to have information about homosexuality available in Asian languages (for parents of gay and bisexual men). This might help to destigmatize homosexuality.

I guess like the one thing that I feel like there has been in terms of my parents there haven't been that many resources for them to access. I think there hasn't been resources where they can go and find out information that is accessible for them. So that has been annoying I think from my perspective because it is difficult to educate them about something that is difficult to talk to them about … so something in Mandarin or in different languages would be useful. (Chinese >5 yrs)

## Discussion

In New Zealand, the burden of HIV/AIDS is experienced disproportionately by MSM, with these men being most likely to be infected (AIDS Epidemiology Group, 2009). Although there has been a rise in the number of HIV diagnoses among gay, bisexual men, and other MSM in New Zealand over the past decade (Saxton, Dickson, Griffiths, Hughes, & Rowden, 2012), the rate of new HIV diagnoses among MSM remains low by international standards (AIDS Epidemiology Group, 2015; Saxton, Dickson, McAllister, Sharples, & Hughes, 2011). Nevertheless this ongoing burden of HIV demands that effective HIV health promotion and prevention initiatives be provided. In particular, understanding the views of particular groups of MSM who may be more vulnerable, such as Chinese and South Asian MSM, will assist in relevant health promotion responses.

The results of our study are that the men interviewed had a strong understanding of the benefits of using condoms for anal sex. They also reported strong recall of HIV health promotion campaigns which seek to influence men's behaviours through a promotion of a single, unequivocal message to always use a condom for anal sex. We know that repeated exposure to these health promotion messages is associated with a greater likelihood of using condoms for anal sex in all partnering contexts (Adams & Neville, 2013). Consequently, there is a strong case for continuing to base HIV health promotion around such social marketing initiatives.

However, in line with other local research, the men in this study did not always report consistent condom use. A range of reasons why this happened were documented including using ways to assess the HIV status of potential partners and the belief that using condoms is unnecessary for men in relationships. These all give opportunities for more tailored interventions to supplement the more generic social marketing messages. One area that may require further attention is in relation to men who were compelled to have sex that they were not comfortable with (i.e., anal sex without condoms). Although there has been some research from New Zealand around this issue, the ways that ethnic minority men are made to feel vulnerable remain unknown. Future initiatives might include ways for such men to be able to more successfully negotiate the use of condoms for anal sex but also to encourage an environment where having respectful and non-coercive sex is everyone's responsibility.

Among the men who discussed testing practices, regular testing was much more likely to have occurred in men who have lived in New Zealand for more than 5 years. Some men who have moved to New Zealand within the last 5 years reported unfamiliarity with testing opportunities and some felt their current sex practices did not warrant participating in regular testing. We note opportunities to enhance men's knowledge of the importance of testing and how to test is an increasing focus of the HIV health promotion work of the NZAF and other organizations (n.d.).

Participants were largely satisfied with Love Your Condom (LYC) social marketing messages. Suggestions to include more Asian content in future health promotion campaigns were provided. As identified above, many participants thought the content incorporated into past and present campaigns was too sexually explicit. The implications for HIV prevention and health promotion programmes are that Chinese, South Asian, and other Asian men should be represented in an appropriate and balanced way. Educational resources could also be developed for family and ethnic communities that demystify and challenge negative perceptions about being gay as these are largely unavailable.

All research has some limitations and as is often the case when conducting research with MSM, participant involvement required men to initiate contact with us. To counter this we advertised and promoted the study widely to ensure the self-selected sample was drawn from the widest potential pool of participants possible. However, men who have no gay community involvement or who do not access gay websites and dating apps were less likely to have seen study recruitment material. This report reflects the views provided by men who were interviewed but does not account for the views of all Chinese and South Asian MSM. Nonetheless, the rich, in-depth, and complex accounts provided by the men allowed for a full exploration of their offered views.

The aim of this research was to identify the way Chinese and South Asian men understand issues related to HIV/STI and health promotion, as well as gaining insight into health promoting behaviours. Our broad sample allowed us to identify links and continuances with local and international research, suggesting that these local results we report may also be relevant outside of the New Zealand situation. In addition, our study contributes to better understanding of the health issues for Chinese and South Asian MSM in a research landscape that has historically focused on the experiences of white men (Clarke, Ellis, Peel, & Riggs, 2010).

## Author Note

Both authors contributed equally to the development of this manuscript.
